# Supplementary figures and images for: Identification and validation of a novel zinc finger protein-related gene-based prognostic model for breast cancer
Source: PeerJ. 2021 Oct 18;9:e12276. doi: 10.7717/peerj.12276 (PMC8530103; doi:10.7717/peerj.12276)

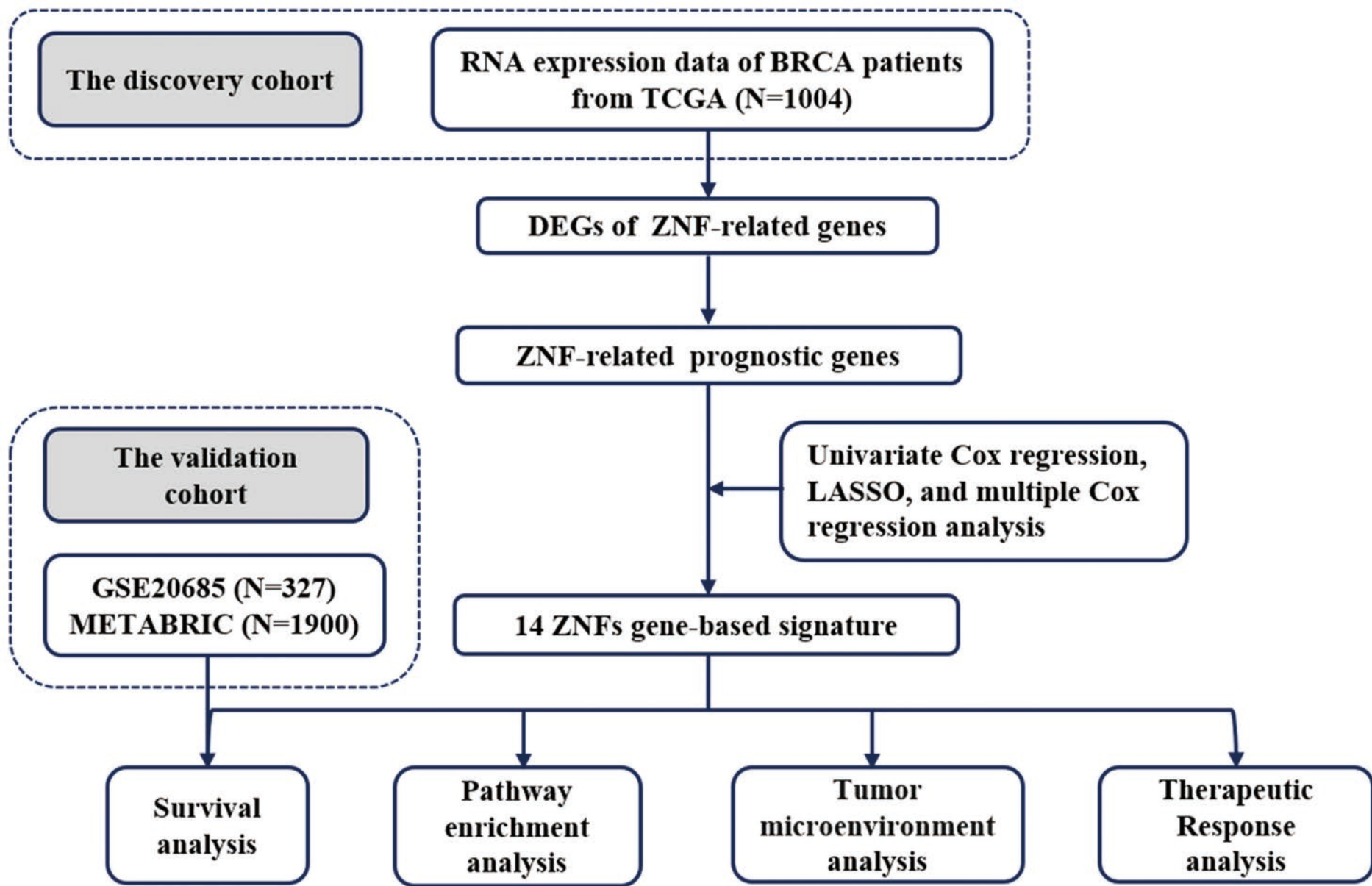

Supplement: Supplemental Information 5 [file peerj-09-12276-s005.pdf]

**A**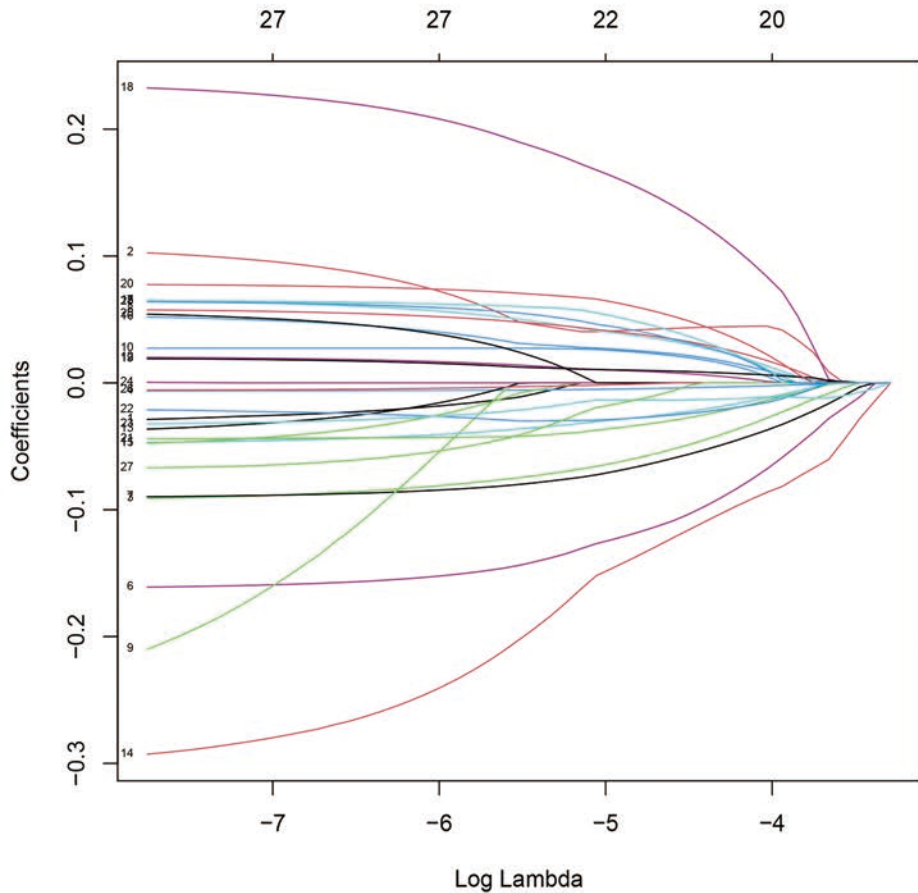**B**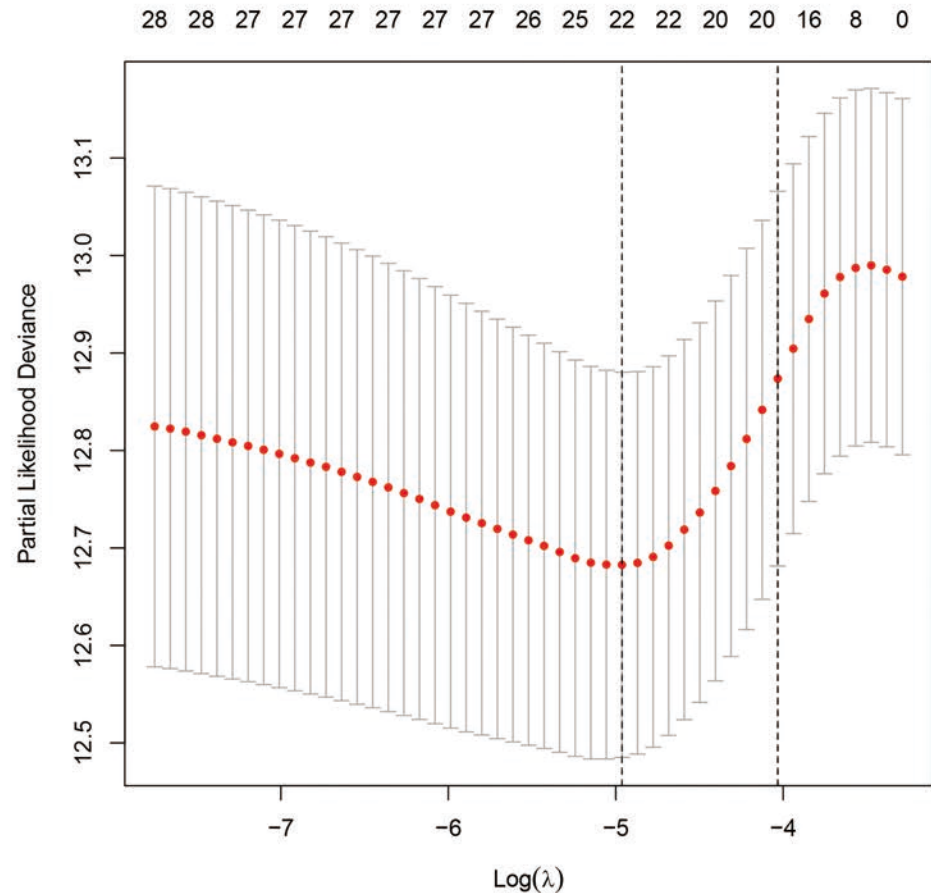

Supplement: Supplemental Information 6 — (A) Ten-fold cross-validation for the coefficients of 320 candidate ZNFs-related genes in the LASSO model. (B) X-tile analysis of the 22 selected ZNFs-related genes. [file peerj-09-12276-s006.pdf]

riskScore 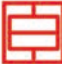 High risk 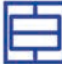 Low risk

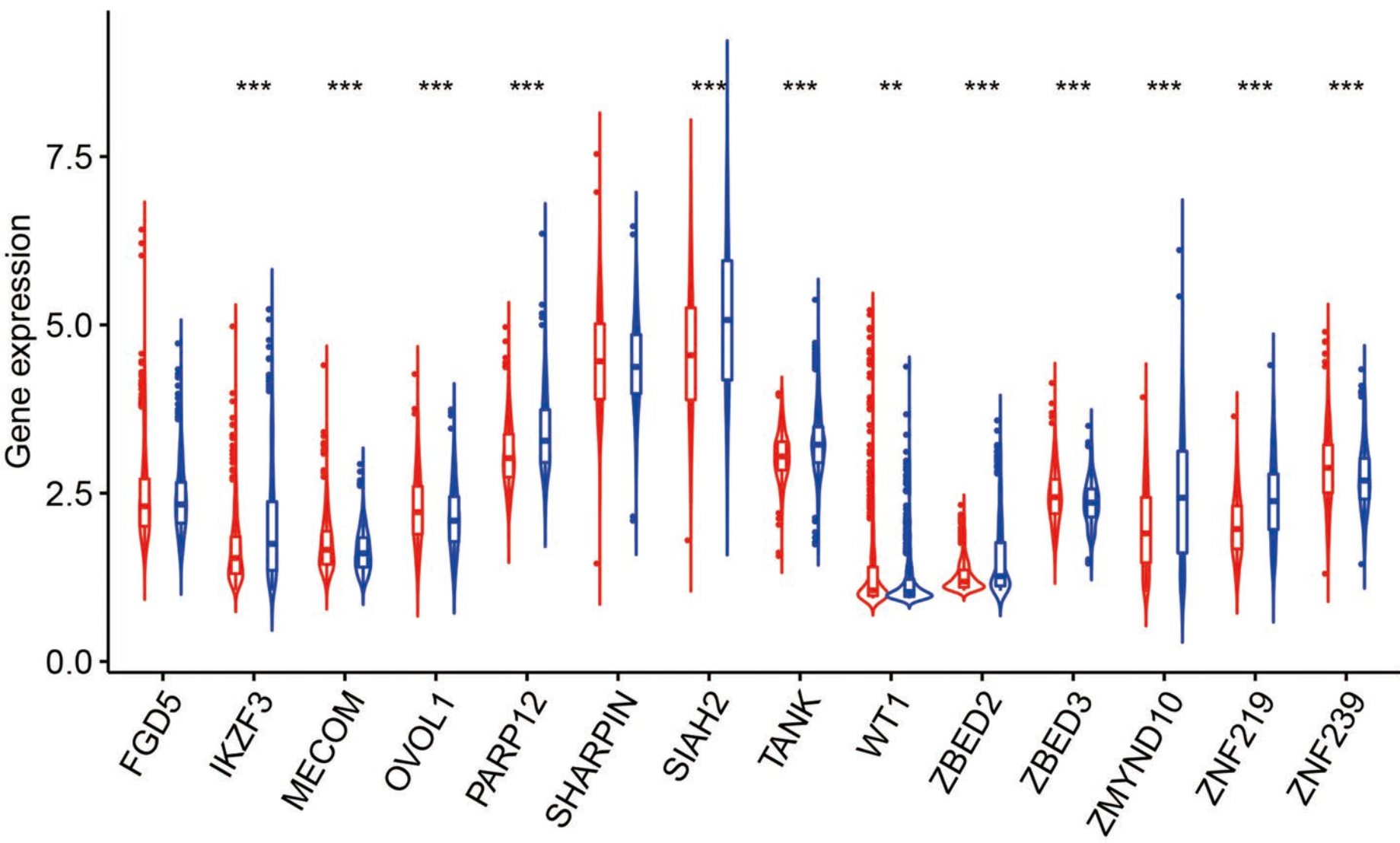

Supplement: Supplemental Information 7 [file peerj-09-12276-s007.pdf]

Survival probability

TNBC

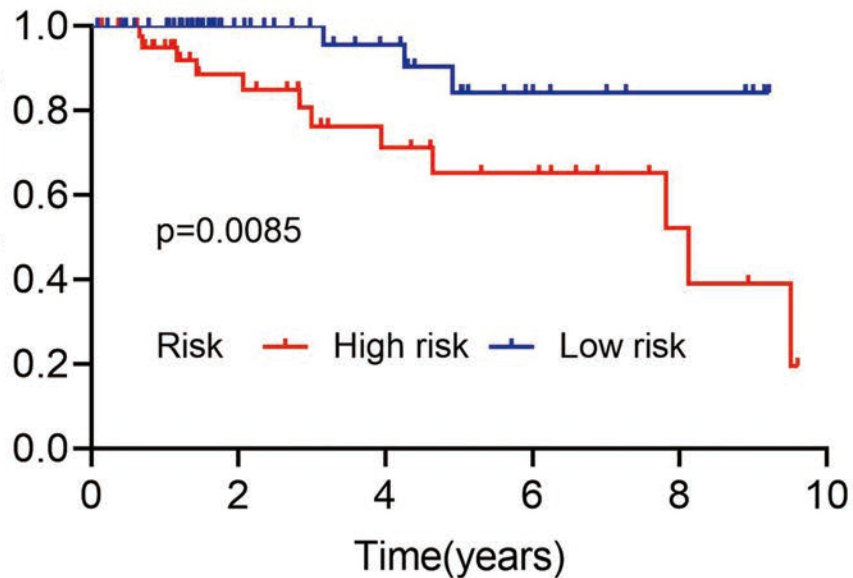

non-TNBC

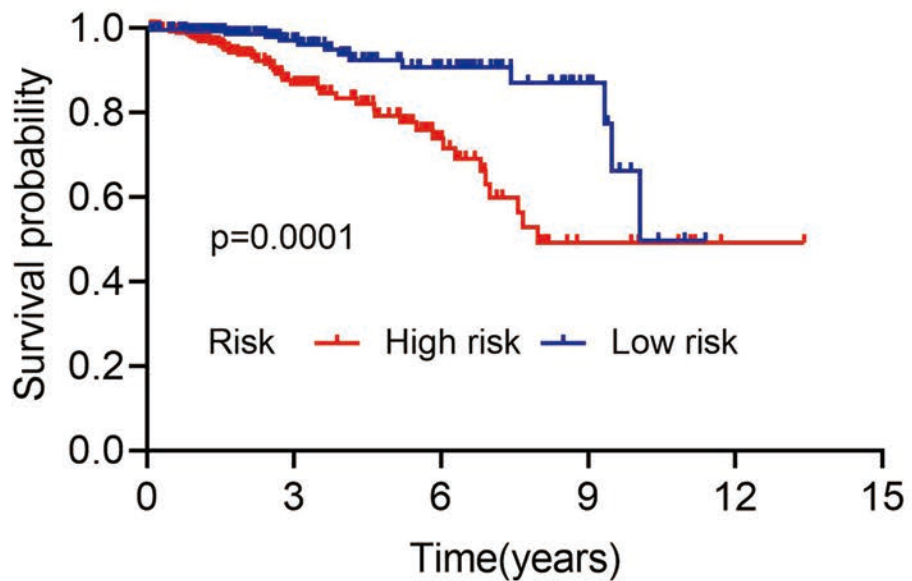

Supplement: Supplemental Information 8 [file peerj-09-12276-s008.pdf]

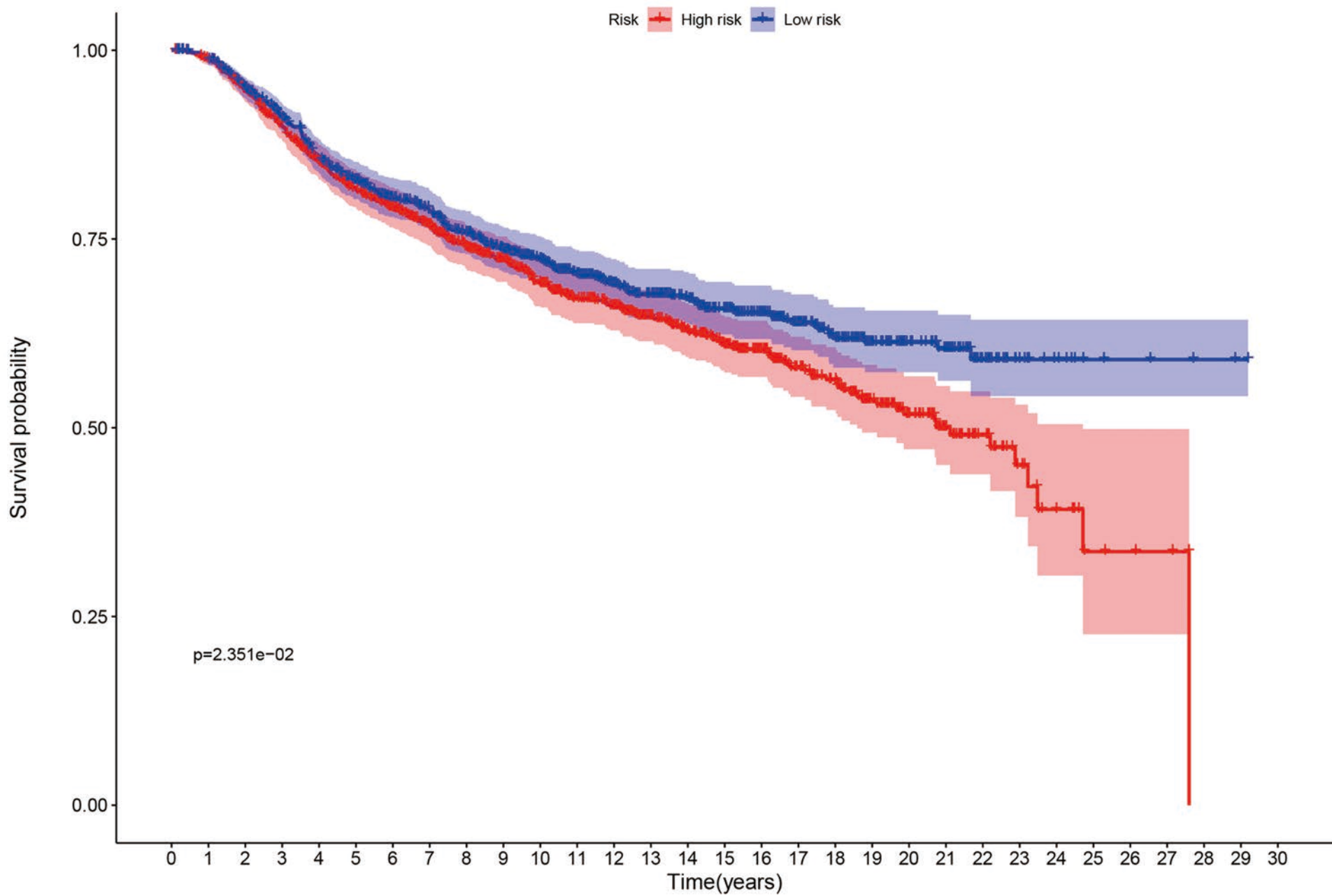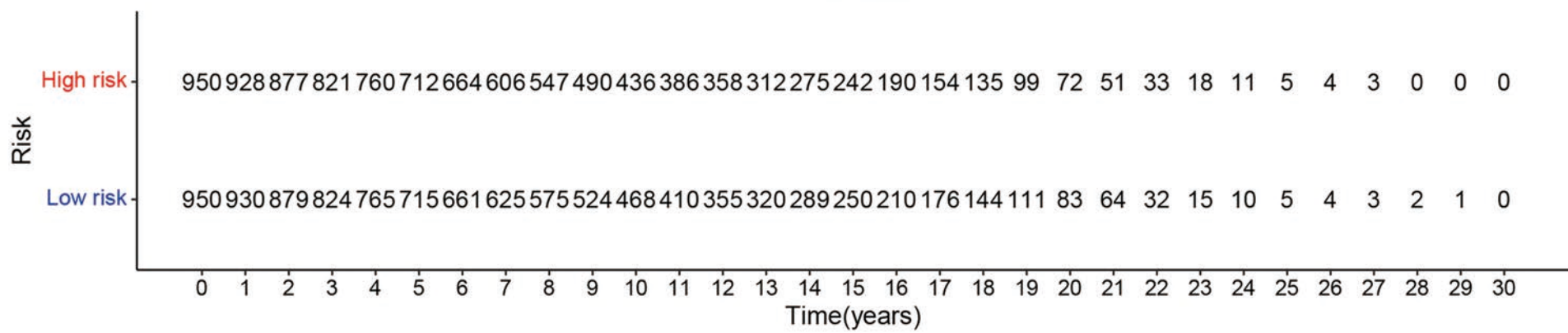

Supplement: Supplemental Information 9 [file peerj-09-12276-s009.pdf]

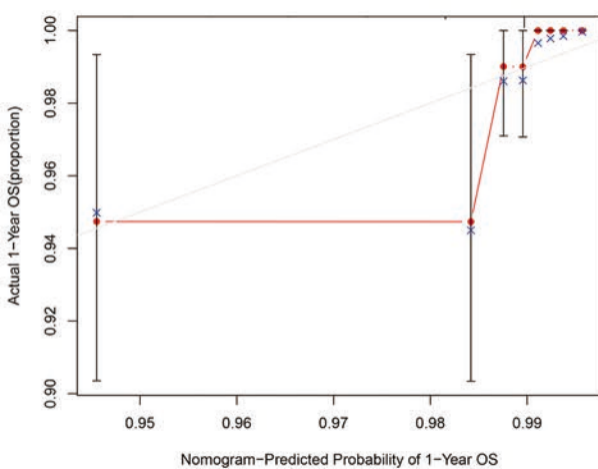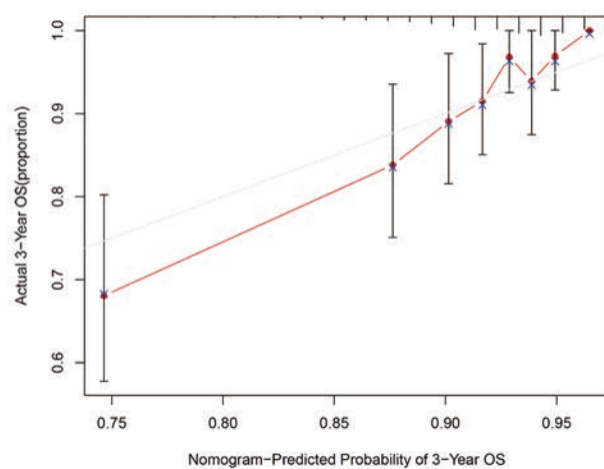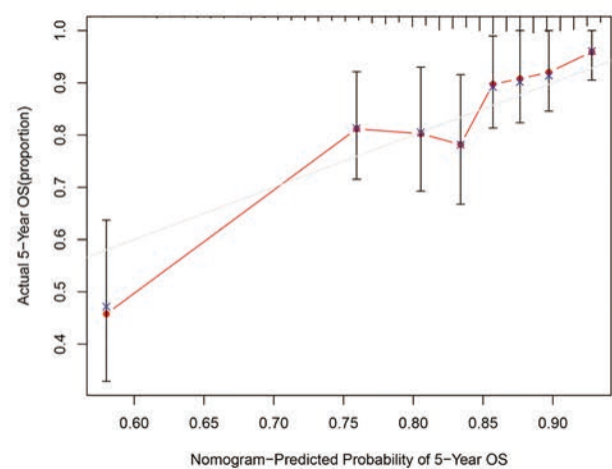

Supplement: Supplemental Information 10 [file peerj-09-12276-s010.pdf]

**A**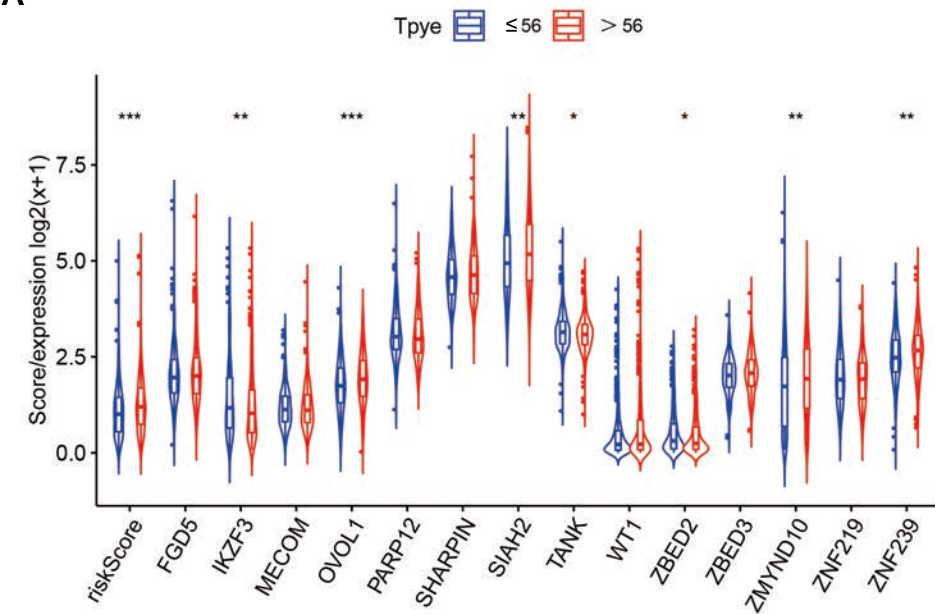**B**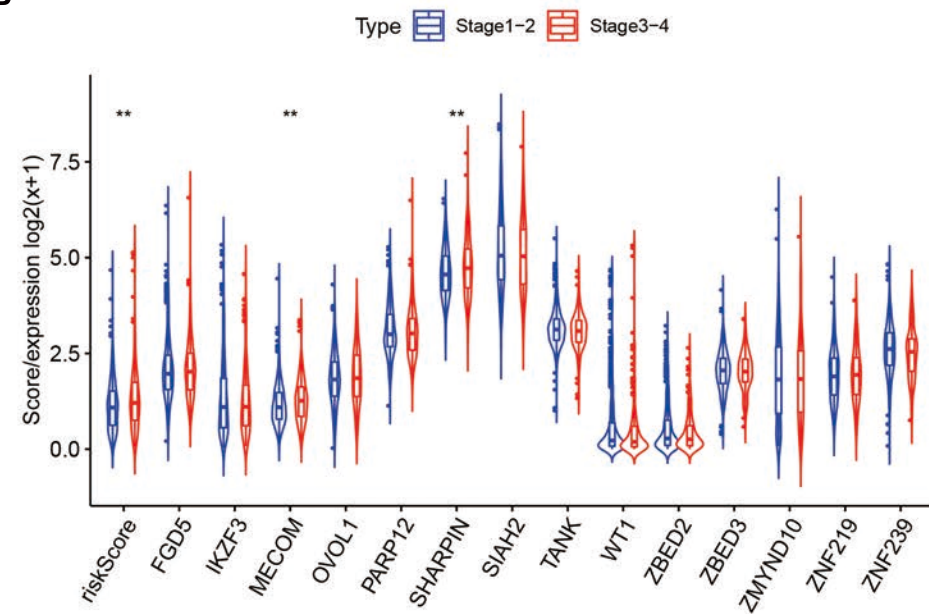**C**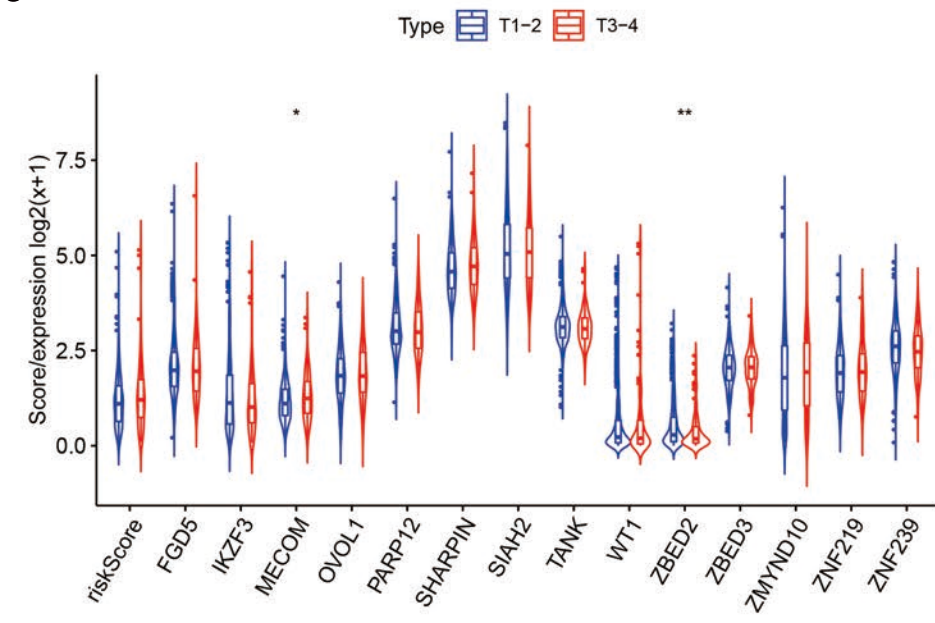**D**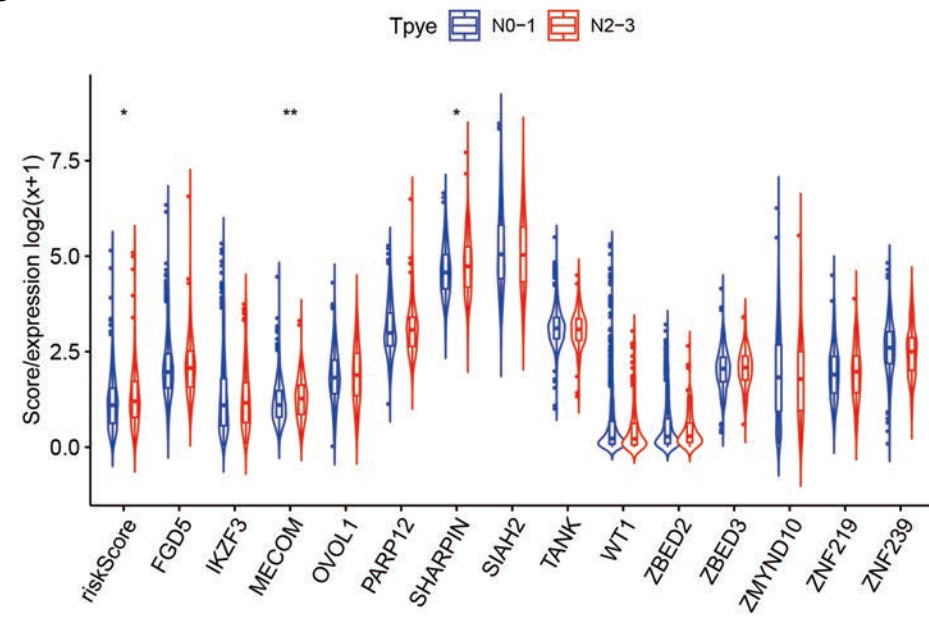

Supplement: Supplemental Information 11 [file peerj-09-12276-s011.pdf]

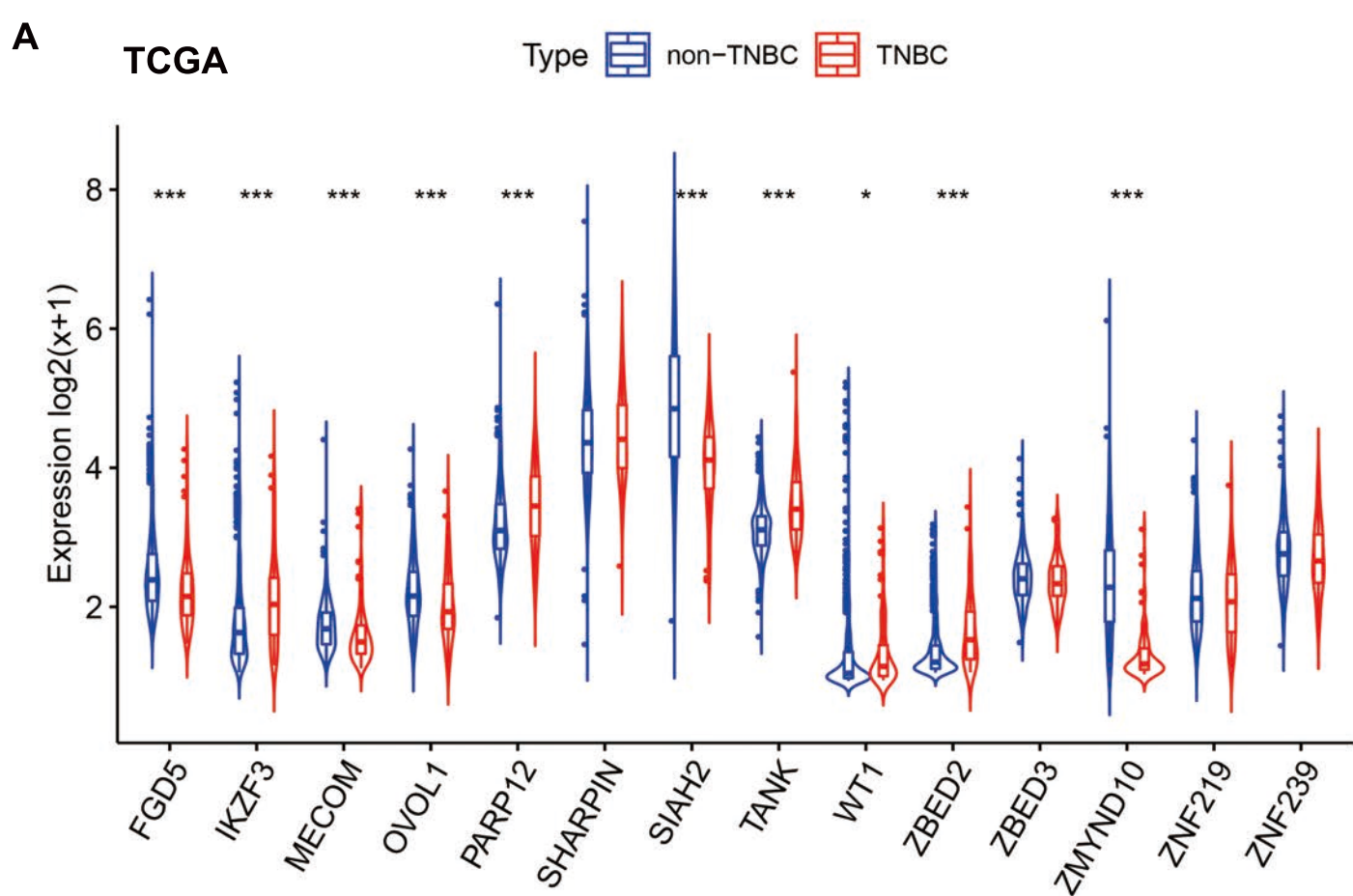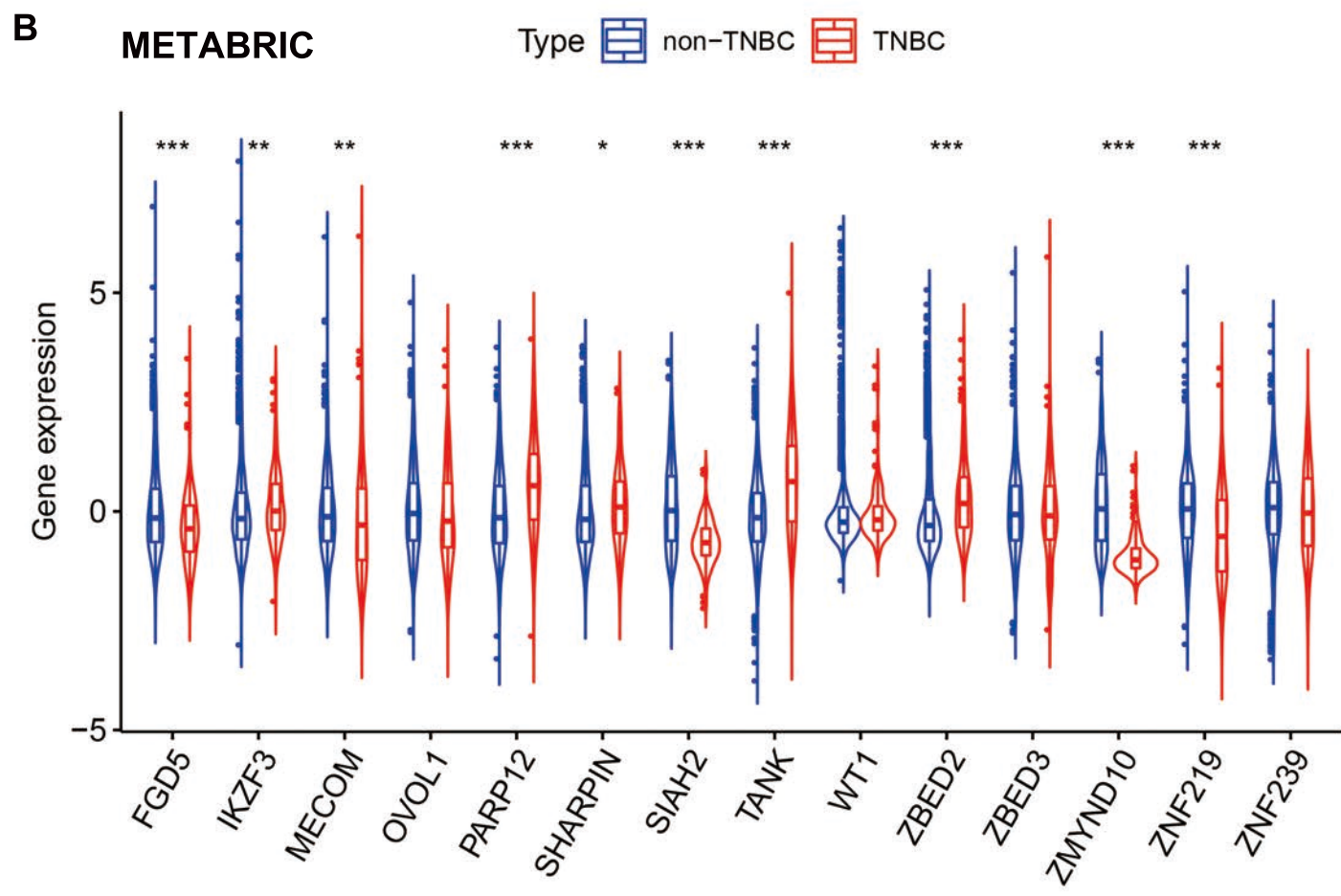

Supplement: Supplemental Information 12 [file peerj-09-12276-s012.pdf]

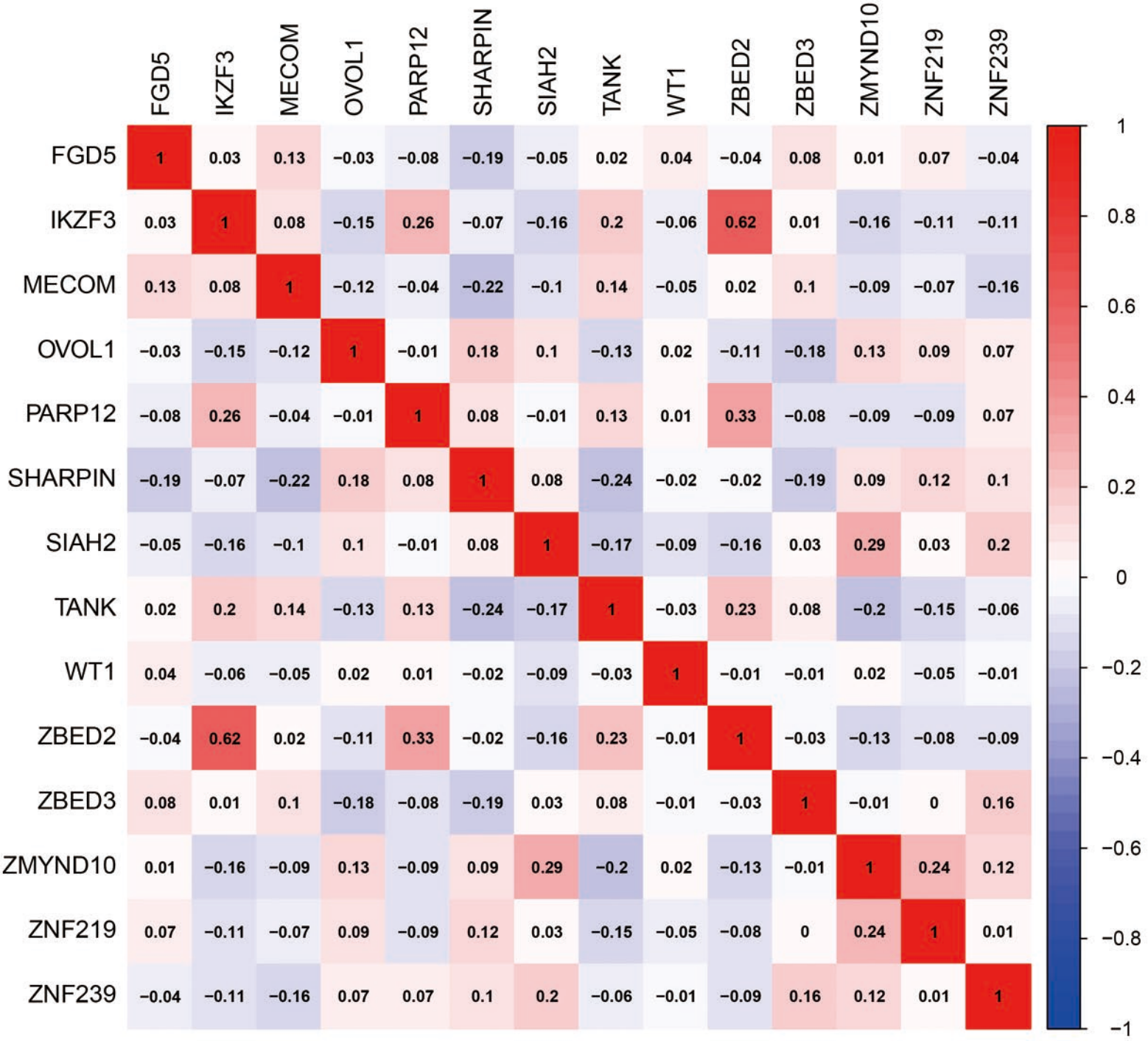

Supplement: Supplemental Information 13 [file peerj-09-12276-s013.pdf]
